# The need for evidence-based orthopedics

**DOI:** 10.4103/0019-5413.30517

**Published:** 2007

**Authors:** Mohit Bhandari, Anil K. Jain

**Affiliations:** Department of Clinical Epidemiology and Biostatistics, McMaster University, Hamilton, Ontario, Canada; * Department of Professor of Orthopedics, University College of Medical Sciences and Guru Teg Bahadur Hospital, Delhi - 95, India

The following symposium marks the commitment of the Indian Journal of Orthopaedics to foster education in the practice of good research practice and the principles collectively known as evidence-based orthopedics.

The term evidence-based medicine (EBM) first appeared in autumn 1990 in a document for applicants to the Internal Medicine residency program at McMaster University that described EBM as an attitude of enlightened skepticism towards the application of diagnostic, therapeutic and prognostic technologies. The most sophisticated practice of EBM requires, in turn, a clear delineation of relevant clinical questions, a thorough search of the literature relating to the questions, a critical appraisal of available evidence and its applicability to the clinical situation and a balanced application of the conclusions to the clinical problem.^1^,^2^ The balanced application of the evidence (i.e., the clinical decision-making) is the central point of practicing evidence-based medicine and involves, according to EBM principles, integration of our clinical expertise and judgment with patients and societal values and with the best available research evidence.

Over the last several years the concepts and ideas attributed to and labeled collectively as EBM have become a part of daily clinical lives and clinicians increasingly hear about evidence-based guidelines, evidence-based care paths and evidence-based questions and solutions. The controversy has shifted from whether to implement the new concepts to how to do so sensibly and efficiently, while avoiding potential problems associated with a number of misconceptions about what EBM is and what it is not (see below). The EBM-related concepts of hierarchy of evidence, meta-analyses, confidence intervals, study design and critical appraisal are so widespread, that surgeons willing to use today's orthopedic literature with understanding have no choice but to become familiar with EBM principles and methodologies.

With the ever-increasing amount of available information, surgeons must consider a shift in paradigm from traditional practice to one which involves question formulation, validity assessment of available studies and appropriate application of research evidence to individual patients.

This symposium highlights articles with a focus on clinical research methodology, common statistical fallacies in research and evidence-based orthopedics. We have several excellent contributions from authors internationally. Along with Dr. Jain, Editor, The Indian Journal of Orthopaedics, we have aimed to develop a practical guide for readers to improve understanding of research methodology and evidence-based approaches in the design and conduct of research studies.
